# Photo Actuation Performance of Nanotube Sheet Incorporated Azobenzene Crosslinked Liquid Crystalline Polymer Nanocomposite

**DOI:** 10.3390/polym11040735

**Published:** 2019-04-23

**Authors:** Meng Bi, Yifan He, Yuchang Wang, Wenlong Yang, Ban Qin, Jiaojiao Xu, Xiuxiu Wang, Binsong Wang, Yinmao Dong, Yachen Gao, Chensha Li

**Affiliations:** 1 Key Laboratory of Functional Inorganic Material Chemistry, Ministry of Education of the People’s Republic of China, Heilongjiang University, Harbin 15008, China; bbmm13704505025@163.com (M.B.); wangyuchang512@163.com (Y.W.); BruceChum941006@163.com (B.Q.); X1143790344@163.com (J.X.); wangxiuxiu053@163.com (X.W.); 2 Key Laboratory of Cosmetic, Beijing Technology and Business University, Beijing 100048, China; heyifan@btbu.edu.cn; 3 Department of Applied Science, Harbin University of Science and Technology, Harbin 150080, China; yangwenlong1983@163.com; 4 Key Laboratory of Chemical Engineering Process and Technology for High-Efficiency Conversion, School of Chemistry and Material Sciences, Heilongjiang University, Harbin 150080, China; 5 Key Laboratory of Electronics Engineering, College of Heilongjiang Province, Heilongjiang University, Harbin 150080, China

**Keywords:** crosslinked liquid crystalline polymer, azobenzene, carbon nanotube, nanocomposite, smart actuator, photo-induced deformation

## Abstract

Crosslinked liquid crystalline polymers (CLCPs) containing azobenzene (AZO-CLCPs) are a type of promising material due to their significance in the design of light-driven smart actuators. Developing AZO-CLCP composites by incorporating AZO-CLCPs with other materials is an effective way of enhancing their practicability. Herein, we report an AZO-CLCP/CNT nanocomposite prepared by the in situ polymerization of diacrylates containing azobenzene chromophores on carbon nanotube (CNT) sheets. The liquid crystal phase structure of CLCP matrix was evidenced by the two-dimensional X-ray scattering. The prepared pure AZO-CLCP films and AZO-CLCP/CNT nanocomposite films demonstrated strong reversible photo-triggered deformation under the irradiation of UV light at 366 nm of wavelength, as a result of photo-induced isomerization of azobenzene moieties in the polymer network. But compared to pure AZO-CLCP films, the AZO-CLCP/CNT nanocomposite films could much more rapidly return to their initial shapes after the UV light irradiation was removed due to the elasticity effect of CNT sheets. The deformation behavior of AZO-CLCP/CNT nanocomposite films under the light irradiation was also different from that of the pure AZO-CLCP films due to the interfacial interaction between a polymer network and CNT sheet. Furthermore, incorporation of a CNT sheet remarkably increased the mechanical strength and robustness of the material. We also used this AZO-CLCP/CNT nanocomposite as a microvalve membrane actuator, which can be controlled by light, for a conceptual device of a microfluidic system. The results showed that this AZO-CLCP/CNT nanocomposite may have great potential in smart actuator applications for biological engineering, medical treatment, environment detection and microelectromechanical systems (MEMS), etc.

## 1. Introduction

Photo-actuated actuators are attractive to researchers since they offer an effective means to couple photonic energy into the actuator structures, and bring advantages of simplified systems with wireless functionality, remote control, green energy, and high-level integration [1,2]. Furthermore, the use of polymer materials to fabricate actuators is beneficial due to their high mechanical flexibility, low weight, excellent corrosion resistance, low-noise operation and easy fabrication features [3]. Therefore, photoactive deformable polymer materials would be especially promising for the development of photo-actuated actuators. Among them, crosslinked liquid crystalline polymers (CLCPs) containing photochromic moieties, such as azobenzene, may represent one of the most studied systems [4,5,6,7,8]. As a crosslinking of liquid crystalline (LC) polymer chains makes a strong correlation between LC mesogens and polymers, while CLCPs combine the characteristics of anisotropic aspect of LC and rubber elasticity of polymer networks. The self-organization function of LC systems and the flexibility stemming from the polymer networks allow for large and reversible anisotropic deformation in response to the applied stimuli [9,10]. CLCPs containing azobenzene (AZO-CLCPs) demonstrate a reversible photo-triggered deformation that is typically induced by isomerization of azobenzene between *trans* and *cis*, this photo-induced isomerization of azobenzene moieties can disrupt or reversely arrange the molecular ordering, and these molecular scale transitions result in remarkable macroscopic deformation [11,12]. Various photo-actuated soft actuators, such as photomechanical cantilevers [13,14], optical pendulum generator [15], plastic motors [16], flexible microrobots [17,18,19,20], micropump [21], microvalve [22], artificial cilia [23], and artificial organs [24], etc, have been made from AZO-CLCPs.

The performances of CLCPs and liquid crystalline elastomers (LCEs), which are lightly cross-linked CLCPs, can generally be modified and improved when they are incorporated with other materials. Researchers have fabricated AZO-CLCP nanocomposites capable of photo-induced deformation upon exposure to near-infrared light or visible light by introducing upconversion nanophosphors into matrices [25,26]. Through the incorporation of polyethylene films, a poly vinyl alcohol matrix or a polyurethane network with CLCPs or LCEs, the materials obtained good forming capabilities, mechanical features and recyclable utilization [16,17,18,27,28,29,30].

Carbon nanotubes (CNTs) and graphene-typed materials have been widely studied for their extraordinary physical properties, such as good absorption in the visible/near-infrared region, high mechanical strengths and conductivities, etc [31]. Therefore, CNTs and graphene-typed materials have been successfully incorporated into CLCPs and LCEs to improve the materials’ mechanical properties, and their unique photo-thermal conversion effect and high thermal conductivities make them efficient nanoscale heaters to generate localized heating upon light irradiation, thereby inducing the LC-to-isotropy phase transition of matrices, and hence the reversible deformation of CLCP or LCE materials [27,28,29,30,32,33,34,35,36,37,38,39,40,41]. CNTs can obviously affect the structure and property of incorporated organic molecules by noncovalent interactions [31], it has been reported that the CNTs with aligned nanostructure could effectively orient the mesogens of CLCPs along the axis of CNTs [42]. The aligned CNTs can also be used to attain a higher photo-mechanical energy conversion efficiency of AZO-CLCPs by amplifying the stress and mechanical force exerted under molecular transition of the azobenzene derivatives [43].

In this work, we report an effective approach to fabricate photodeformable AZO-CLCP/CNT nanocomposite composed of a AZO-CLCP layer and CNT sheet. Homogeneous alignment of LC mesogens was achieved by the aligning-structured polyimide layer coated on glass substrate [44] and the nanocomposite film formed by the in situ photo polymerization of precursor mixture on CNT sheet. In the AZO-CLCP/CNT nanocomposite films, the AZO-CLCP layers functioned as photoresponsive parts that provided photo actuation performance for the materials, and the CNT sheets as photoinert substrates that provided good film-formation and mechanical properties. The designed structure and composition endowed the AZO-CLCP/CNT nanocomposite with salient features, including easy fabrication, low cost, high mechanical properties, good robustness and photo actuation performance. Compared to the pure AZO-CLCP films, the AZO-CLCP/CNT nanocomposite films could not only deform with the similar responsive speed under the irradiation of UV light at 366 nm of wavelength, but also could much more rapidly return to their initial shapes after the UV light irradiation was removed due to the elasticity effect of CNT sheets. Furthermore, the AZO-CLCP/CNT nanocomposite films moved in a different photodeformable direction compared to the pure AZO-CLCP films, which should be caused by the interfacial interaction between the polymer network and the CNT sheet [45]. The photo actuation performance was influenced by the content of azobenzene moieties contained in AZO-CLCP matrix, the optimum photo actuation performance could be achieved in a suitable content of azobenzene moieties. Based on the superior performances demonstrated by the AZO-CLCP/CNT nanocomposite films, we used this nanocomposite as the microvalve membrane actuator for a conceptual device of a microfluidic system. Experiment indicated that the switching of the microvalve could be precisely controlled by light. These results showed that this AZO-CLCP/CNT nanocomposite may have great potential in smart actuator applications for biological engineering, medical treatment, environment detection and microelectromechanical systems (MEMS), etc.

## 2. Experimental Procedures

### 2.1. Materials Preparation

The azobenzene-contained diacrylate LC monomer “4,4′-bis[1 1-(acryloyloxy)nonyloxy]azobenzene (DA11AB)” and diacrylate LC monomer “1,4-bis[4-(9-acryloyloxynonyloxy)benzoyloxy]-2-methylbenzene (C9A) ” were synthesized by referencing the previous reported work [19]. The initiator “Irgacure 784” was purchased from commercial supplier, Sigma-Aldrich (St Louis, MO, USA), and used as received. The CNT sheets with a thickness of about 10 µm were obtained from Xianfeng Nano-Tech (Nanjing, China).

The AZO-CLCP films were prepared by the photopolymerization of DA11AB and C9A, containing 2 mol % of initiator (Figure S1). The photopolymerization was carried out in a glass cell (as the reaction chamber) with about 20 µm thick gap. The two inner surfaces of the cell had been coated with a polyimide alignment layer that had been rubbed to align the LC mesogens. The powdered precursor was a mixture of DA11AB and C9A with a designed molar ratio, and the initiator was heated to 110 °C to melt. The liquid precursor mixture was injected into the cell, and then the temperature of the cell was gradually decreased to 93 °C at which the liquid precursor mixture was in a LC phase. The precursor mixture in LC phase was cross-linked by photopolymerization at >540 nm (3 mW·cm^−2^) with a high-pressure mercury lamp (CHANGTUO CHF-XM250, Beijing, China) through a glass filter for 3 h. As the polymerization completed, the cell was opened, and the AZO-CLCP film of about 20 µm thickness was removed from the cell with a cutter. The AZO-CLCP/CNT nanocomposite films were prepared via the same procedure, but the photopolymerization was carried out in the glass cell with a CNT sheet sandwiched between the two inner surfaces (Figure S2). The prepared AZO-CLCP films with the molar ratio of DA11AB and C9A being 1:9, 2:8, 4:6 and 6:4 respective are designated as F10, F20, F40 and F60 respectively. The prepared AZO-CLCP/CNT nanocomposite films with the molar ratio of DA11AB to C9A being 1:9, 2:8, 4:6 and 6:4 respective are designated as CF10, CF20, CF40 and CF60 respectively.

### 2.2. Characterization Methods

The chemical structures of the synthesized DA11AB and C9A were measured by the way of nuclear magnetic resonance (NMR) spectra cooperated with mass spectra (MS). ^1^H NMR spectra were recorded in CDCl_3_ solutions with a Bruker Mercury300 spectrometer (Billerica, MA, USA) using tetramethylsilane (TMS) as the internal reference. Mass spectra (MS) were taken with a Bruker Daltonics Ultraflex III MALDI TOF/TOF Mass Spectrometer (Billerica, MA, USA). The morphology and structure of the CNT sheet and AZO-CLCP/CNT nanocomposite were examined by scanning electron microscopy (SEM; Zeiss LEO 1530, Thornwood, NY, USA). The mesomorphic properties of AZO-CLCP films were observed by using polarizing optical microscopy (POM, Nikon Instruments, SMZ 1500, Melville, NY, USA). The mesomorphic properties of AZO-CLCP/CNT nanocomposite films were examined by the two-dimensional X-ray scattering (2D-WAXS) experiments which were performed by a Bruker/Siemens Hi-Star 2D X-ray Diffractometer (Billerica, MA, USA) with a monochromatic CuKalpha point source (0.8 mm). A 366 nm UV-LED (Omron, ZUV-C30H, 100 mW cm^−2^, Shenzhen, China) was used as the light source for photo-actuated deformation experiments. A universal material mechanical analyzer (CMT-10, LG Company, Jinan, China) with a pair of tension clamps was employed to measure the mechanical properties or photomechanical properties of the AZO-CLCP/CNT films and AZO-CLCP/CNT nanocomposite films, the two ends of the tested material were gripped by the two clamps. The environment temperature of experiments was 20 °C.

## 3. Results and Discussions

**Figure 1:**
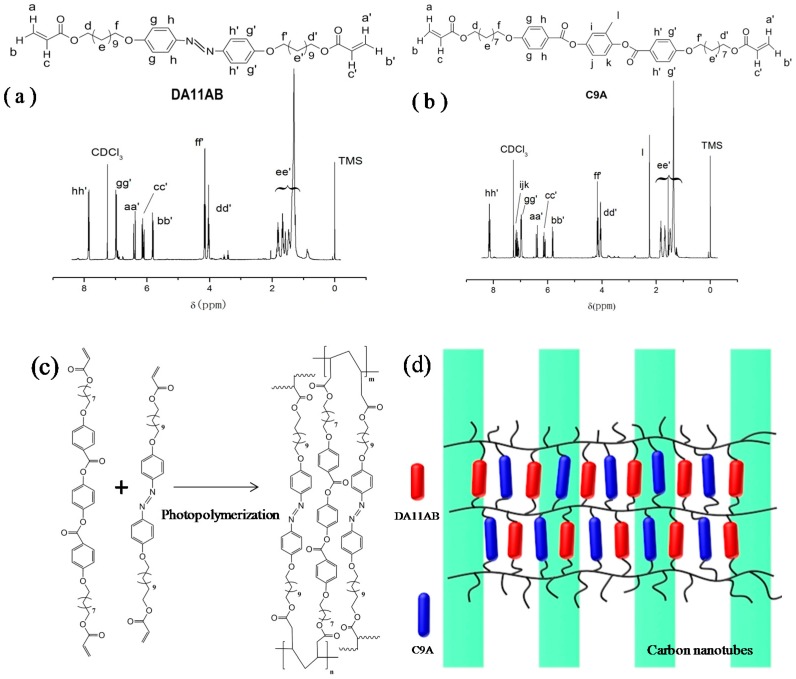
(**a**) Chemical structure and ^1^H NMR spectrum of DA11AB; (**b**) Chemical structure and ^1^H NMR spectrum of C9A; (**c**) Illustration of the formation of AZO-CLCP through photopolymerization of DA11AB and C9A; (**d**) Illustration of AZO-CLCP/CNT nanocomposite.

The chemical structures and ^1^H NMR spectra of DA11AB and C9A are shown in Figure 1. The data of ^1^H NMR and MS measurements of DA11AB and C9A are in the Support Information. From the measurements of ^1^H NMR and MS, the chemical structures of the synthesized monomers are consistent with the standard chemical structures of DA11AB and C9A.

The free radical photopolymerization reaction process of AZO-CLCP formation is shown in Figure 1c. The olefinic bonds of acrylate groups were opened by the initiation of initiator, and then polymerized through chain addition to form crosslinked network. The structure of AZO-CLCP/CNT nanocomposite was constituted by the crosslinked network and CNTs, as shown in Figure 1d.

**Figure 2:**
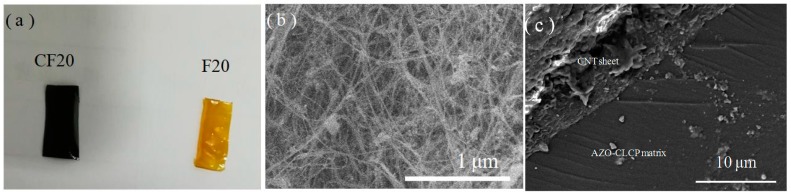
(**a**) A photo image of the prepared AZO-CLCP film (F20) and AZO-CLCP/CNT nanocomposite film (CF20). Size of the films: 12 mm × 6 mm × 20 μm; (**b**) A SEM image of the CNT sheet; (**c**) A SEM image of the cross section of AZO-CLCP/CNT nanocomposite film (CF20).

Figure 2a, a photo image of the prepared AZO-CLCP film and AZO-CLCP/CNT nanocomposite film, shows that the AZO-CLCP film was transparent yellow film, and the AZO-CLCP/CNT nanocomposite film was opaque black film. Figure 2b, a SEM image of the CNT sheet, shows that the CNT sheet had a dense network structure formed by the CNTs with a uniform diameter of about 20 nm. Figure 2c, a SEM image of the cross section of an AZO-CLCP/CNT nanocomposite film, shows that the AZO-CLCP matrix and CNT sheet were compactly integrated.

**Figure 3:**
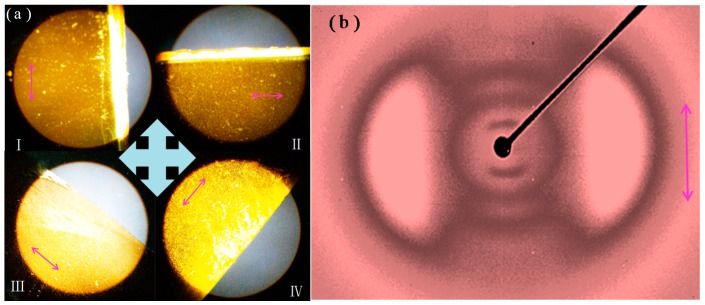
(**a**) POMs of the AZO-CLCP film(F20): (I) The rubbing direction is parallel to the vertical polarization direction; (II) The rubbing direction is parallel to the horizontal polarization direction; (III) The angle between the rubbing direction and the polarization direction of either polarizer is −45°; (IV) The angle between the rubbing direction and the polarization direction of either polarizer is 45°. Inserted crossarrows illustrate the polarization directions of the two polarizers, and the pink arrows illustrate the rubbing directions; (**b**) 2D-WAXS pattern of the AZO-CLCP/CNT nanocomposite film (CF20). Inserted pink arrow illustrates the rubbing direction.

The polymerization was carried out in a cell that was precoated with rubbed polyimide alignment layers. The alignment layer assisted to align the LC mesogens when the precursors were melted under heating so that they could be polymerized in an aligned state. The POM was used to evaluate the alignment effect of the LC mesogens in AZO-CLCP film by measuring the transmittance of a probe light through two crossed polarizers with an AZO-CLCP film between them. As shown in Figure 3a, the highest transmittance appears when the angle between the rubbing direction of the alignment layer and the polarization direction of either polarizer is ±45°, while the lowest appears when the rubbing direction is parallel to one of the polarization directions. Periodic changes of dark and bright images were observed by rotating the sample with an interval of 45°. The POM observations of the AZO-CLCP films with different molar ratio of DA11AB to C9A exhibited a consistent result, which proved an LC alignment structure, and that the LC mesogenic units were well aligned along the rubbing directions of the alignment layers. The alignment effect of the LC mesogens in AZO-CLCP/CNT nanocomposite films cannot be measured by POM due to their opacity. Figure 3b shows the 2D-WAXS pattern of an AZO-CLCP/CNT nanocomposite film measured with the incident beam perpendicular to the film surface, the azimuthal intensity maxima at wide-angle reflection indicate the alignment structure of LC mesogens in a CLCP matrix [35,41]. The 2D-WAXS patterns of the AZO-CLCP/CNT nanocomposite films with different molar ratio of DA11AB to C9A also exhibited consistent result that the locations of the wide-angle reflection were orthogonal to the rubbing directions of the alignment layers, thus further confirming the LC alignment structure of CLCP matrices, and that the LC mesogenic units were well aligned along the rubbing directions of the alignment layers.

**Figure 4:**
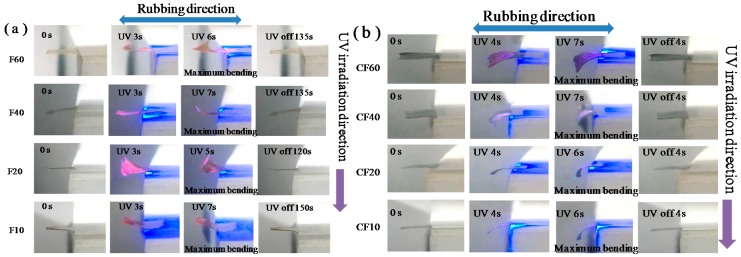
Photographs that exhibit the photoinduced deformation behavior of the AZO-CLCP films (**a**), and the AZO-CLCP/CNT nanocomposite films (**b**). Size of the films: 12 mm × 6 mm × 20 μm. The wavelength and intensity of the used UV light was 366 nm and 1.5 mW/cm^2^ respective. The room lamplight kept turning on state constantly.

The prepared AZO-CLCP films and AZO-CLCP/CNT nanocomposite films demonstrated photoinduced reversible deformation behaviors. As shown in Figure 4, the partially free-standing AZO-CLCP films and AZO-CLCP/CNT nanocomposite films were put on the glass substrates and in flat initial state, and then a normal irradiation of 366 nm UV light with the intensity of 1.5 mW/cm^2^ was performed.

Figure 4a shows that the AZO-CLCP films bent toward the irradiation direction of incident UV light along the rubbing direction. They bent to the maximum extents after being exposed to UV light for 5 to 7 s, and no further bending was observed upon further UV irradiation. Additionally, the bent AZO-CLCP films completely reverted to the initial flat state in 2 to 2.5 min after the UV light was switched off. This photoinduced bending and unbending behavior could be repeated by alternate on-off switch of a UV light. The photo actuation of AZO-CLCP films should be derived from the structure changes in materials. UV light irradiation induced the *trans–cis* isomerization of azobenzene moieties, which resulted in a reduction in the size of azobenzene moieties and the alignment order of LC mesogens, and subsequently in a contraction of the polymer network along the alignment direction [1,5]. The majority of the UV energy was absorbed by the azobenzene moieties near the surface due to the strong UV absorption of azobenzene moieties [46]. Thus, this anisotropic contraction occurred only at the near-surface regions of the AZO-CLCP films, causing the bending toward the incident UV light and along the alignment direction, which was the rubbing direction. After the UV light was switched off, the AZO-CLCP films were only exposed to the irradiation of room lamplight, which constantly kept turning on state. The visible light above 530 nm contained in the room lamplight induced the *cis*–*trans* isomerization of azobenzene moieties [1,5], and hence the polymer network reverted to its initial structure, resulting in the reversion of initial flat state of AZO-CLCP films.

Figure 4b shows that the AZO-CLCP/CNT nanocomposite films bent beyond the irradiation direction of incident UV light along the rubbing direction. They bent to the maximum extents after being exposed to UV light for 6 to 7 s, and completely reverted to the initial flat state in 4 s after UV light was switched off. This photoinduced bending and unbending behavior also could be repeated by an alternate on-off switch of UV light. The essential mechanism of photo actuation of CLCP/CNT nanocomposite films was similar to that of the AZO-CLCP films, which was due to the *trans–cis* isomerization of azobenzene moieties induced by the UV irradiation. However, the AZO-CLCP/CNT nanocomposite films behaved in an opposite photodeformable way compared to the AZO-CLCP films, which should be caused by the interfacial interaction between the CLCP network and CNT sheet [45]. The mesogens in the interfacial layers between CLCP network and CNTs’ surfaces were preferentially oriented parallel to CNTs’ surfaces in order to maximize the π–π interaction. The photo-isomerization of azobenzene moieties reduced the π–π interaction energy and broke the arrangement of interfacial layers, some phenyl groups of azobenzene moieties were bent perpendicular to the CNTs’ surfaces to form the edge-to-face π–π stacking conformation. This structure change in interfacial layers caused a great contraction of the interphase region between the CLCP network and CNTs’ surfaces whose extent was larger than that of the bulk region of CLCP network, thus the AZO-CLCP/CNT nanocomposite films indicated a deformation behavior of bending beyond the incident UV light and along the alignment direction, which was the rubbing direction. After the UV light was switched off, the room lamplight, which constantly kept on, induced the *cis*–*trans* isomerization of azobenzene moieties, and hence the CLCP network reverted to its initial structure, resulting in the reversion to the initial flat state of the AZO-CLCP/CNT nanocomposite films. Comparing Figure 4a,b, it indicates that the AZO-CLCP/CNT nanocomposite films could not only bend with a similarly rapid speed as the AZO-CLCP films could under the irradiation of UV light, but also could much more rapidly return to their initial shapes after the UV light irradiation was removed. The reason for this should be that the elasticity effect of CNT sheet brought in a tensile stress along the alignment direction, which accelerated the *cis*–*trans* isomerization process of azobenzene moieties.

It is interesting to note that the maximum bending extents of films varied with different molar ratios of DA11AB to C9A. F20 demonstrated the highest maximum bending extent among the AZO-CLCP films while CF20 demonstrated the highest maximum bending extent among the AZO-CLCP/CNT nanocomposite films. The reason for this should be that the films with different molar ratio of DA11AB to C9A had different amounts of azobenzene moieties. A low content of azobenzene moieties would create weak contraction actuation under UV irradiation, resulting in a low bending extent of the film. Higher content of azobenzene moieties would mean a greater percentage of UV energy being absorbed in the near-surface region of the film. For an AZO-CLCP film, a too high content of azobenzene moieties could also weaken the contraction actuation under UV irradiation due to the depth of the region in which contraction occurred being too low, resulting in a lower bending extent of the film. For an AZO-CLCP/CNT nanocomposite film, a too high content of azobenzene moieties would result in the contraction extent of interphase region being seriously decreased since a very low percentage of UV energy could be absorbed by this region, thus the bending extent of the film under UV irradiation would also be lowered. Therefore, a suitable content of azobenzene moieties is needed to create the highest deformation effect of the film under UV irradiation. It revealed from Figure 4 that the molar ratio of 2:8 of DA11AB to C9A could create the highest photoinduced deformation effect both for AZO-CLCP films and for AZO-CLCP/CNT nanocomposite films.

**Figure 5:**
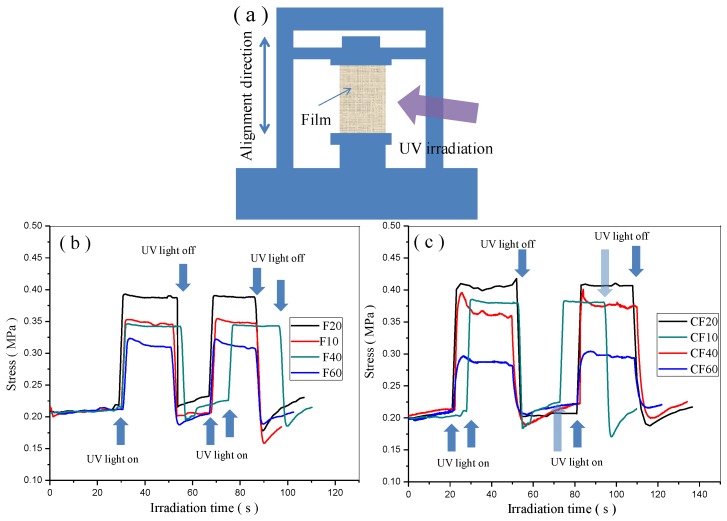
(**a**) Schematic illustration of the experimental setup for measuring the photoinduced stresses of AZO-CLCP films and AZO-CLCP/CNT nanocomposite films under irradiation of UV light. Size of the films: 12 mm × 6 mm × 20 μm. The wavelength and intensity of the used UV light was 366 nm and 1.5 mW/cm^2^ respective. The room lamplight kept turning on state constantly; (**b**) Photoinduced stresses of the AZO-CLCP films; (**c**) Photoinduced stresses of the AZO-CLCP/CNT nanocomposite films.

The photoinduced force is one of the important factors for photo-actuated materials. The photomechanical properties of the prepared AZO-CLCP films and AZO-CLCP/CNT nanocomposite films were quantitatively studied by using a universal material mechanical analyzer, as shown in Figure 5a. Every film for photomechanical measurement was fixed at both ends and an initial tensile stress of 0.2 MPa was used to the film to make it in a flat state. When UV light irradiated on the films’ surfaces, the bending stresses of the films rapidly increased. After the UV light was turned off, the films’ stresses rapidly returned to their initial states, as shown in Figure 5b,c. The photoinduced stresses of the films could be stable after repeating cycles of UV light irradiation. In addition, F20 demonstrated the highest photoinduced stress among the AZO-CLCP films while CF20 demonstrated the highest photoinduced stress among the AZO-CLCP/CNT nanocomposite films, thus revealing that the molar ratio of 2:8 of DA11AB to C9A created the best photomechanical properties.

The AZO-CLCP films and AZO-CLCP/CNT nanocomposite films showed anisotropic mechanical properties due to the anisotropic network structure of AZO-CLCP matrices. The tensile strengths were measured both parallel and perpendicular to the alignment direction by using the universal material mechanical analyzer (Figure S3), and the results are summarized in Table 1. The stress-strain curves of some AZO-CLCP films and AZO-CLCP/CNT nanocomposite films are in the Supporting Information (Figure S4). The films demonstrated better mechanical properties in the parallel direction than in the perpendicular direction. On the other hand, the tensile strengths of the AZO-CLCP/CNT nanocomposite films were obviously higher than those of the AZO-CLCP films, this should be attributed to the reinforcement effect of the CNT sheet.

**Figure 6:**
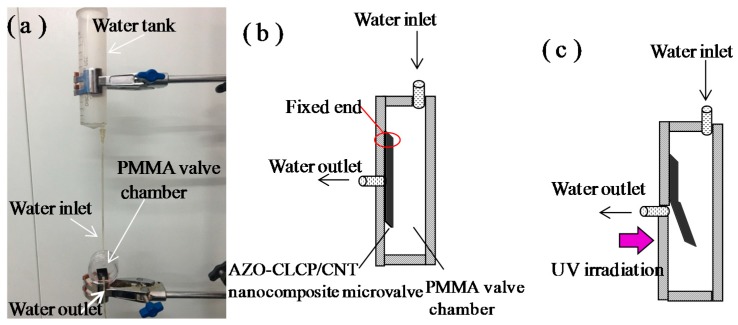
(**a**) A Photograph of experimental setup of the conceptual device of microfluidic system. A CF20 with the size of 12 mm × 6 mm × 20 μm was used as the microvalve actuator; (**b**) A Scheme of the PMMA valve chamber with the AZO-CLCP/CNT nanocomposite microvalve actuator set in it without the UV irradiation; (**c**) A Schematic illustration of the switching on process of the microvalve under irradiation of UV light.

The superior photo actuation performances, well mechanical properties and robustness feature demonstrated by the AZO-CLCP/CNT nanocomposite films, are promising for application in light-driven smart actuators. Considering the optimum photo actuation performance of CF20, we demonstrated it as a light-controlled microvalve membrane actuator for a conceptual device of microfluidic system, the experimental setup is shown in Figure 6a. Inside the polymethyl methacrylate (PMMA) valve chamber, the flat AZO-CLCP/CNT nanocomposite film initially covers the valve seat surface to close the valve, so the fluid in inlet channel cannot access the outlet channel, as illustrated in Figure 6b. Since PMMA has good transmission of light, the light beam can be manipulated outside freely to control the membrane actuator. As illustrated in Figure 6c, when irradiating the film with UV light from the outside of the PMMA chamber the valve will be opened, since the film bends beyond the UV irradiation. When the UV light is removed, the film will revert to an initial flat state under the irradiation of room lamplight and thus the valve will be closed.

**Figure 7:**
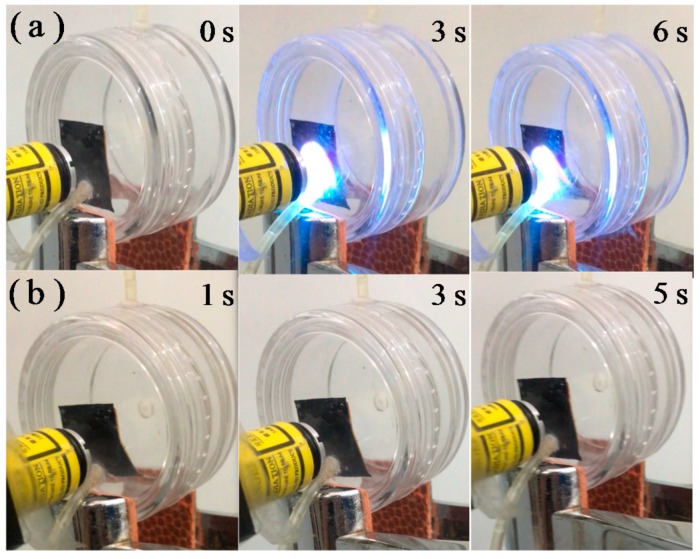
Photographs that exhibit the simulated switching on process of AZO-CLCP/CNT nanocomposite microvalve under irradiation of UV light (**a**) and switching off process of AZO-CLCP/CNT nanocomposite microvalve after the UV source was switched off (**b**). A CF20 with the size of 12 mm × 6 mm × 20 μm was used as microvalve actuator. The wavelength and intensity of the used UV light was 366 nm and 1.5 mW/cm^2^ respective. The room lamplight was kept on consistently.

The microvalve membrane actuator demonstrated a sensitive function of opening or closing the valve under light regulation. As shown in Figure 7 and Movie S1, under UV irradiation, the microvalve membrane actuator rapidly bent beyond the UV incidence and the valve was opened within several seconds. When the UV light was removed, the microvalve membrane actuator rapidly reverted to an initial flat state and the valve was also closed within several seconds. The opening and closing processes could be repeated for more than a hundred cycles without obvious performance decay.

## 4. Conclusions

We report an effective approach to fabricate photo-actuated AZO-CLCP/CNT nanocomposite films by incorporating CNT sheets into AZO-CLCP matrices through a facile melting process and in situ photo polymerization. Compared with the pure AZO-CLCP films, the AZO-CLCP/CNT nanocomposite films demonstrated much improved mechanical properties, robustness feature and photo actuation performances.

The AZO-CLCP/CNT nanocomposite films could not only deform with similar rapid speeds as the pure AZO-CLCP films could under the irradiation of UV light, but also could much more rapidly return to their initial shapes after the UV light was removed. The AZO-CLCP/CNT nanocomposite films also indicated a different photodeformable direction relative to the pure AZO-CLCP films due to the interfacial interaction between the polymer network and CNT sheet. The optimum photo actuation performance could be achieved when the AZO-CLCP matrix contained a suitable content of azobenzene moieties.

Utilizing the AZO-CLCP/CNT nanocomposite film which possessed the optimum photo actuation performance, we demonstrated a light-controlled microvalve membrane actuator for a conceptual device of a microfluidic system. It effectively realized the function of a microvalve. The valve could be sensitively opened or closed by light regulation, and the opening and closing processes could be repeated for many times without obvious decay. Our work sheds significant insight for the development of high-performance actuation materials and their applications in driving smart actuators and microrobots, etc.

## Figures and Tables

**Table 1:** Mechanical properties of the AZO-CLCP films and AZO-CLCP/CNT nanocomposite films measured parallel (‖) and perpendicular (┴) to the alignment direction.

Tensile Strength (MPa)								
	F10	F20	F40	F60	CF10	CF20	CF40	CF60
‖	14.62	14.74	14.81	14.86	32.51	32.64	32.71	32.79
┴	7.51	7.62	7.68	7.74	20.42	20.53	20.71	20.82

## Supplementary Materials

The following are available online at https://www.mdpi.com/2073-4360/11/4/735/s1, Figure S1: A schematic illustration of the preparation process of AZO-CLCP film, Figure S2: A schematic illustration of the preparation process of AZO-CLCP/CNT film, Figure S3: Schematic illustration of the experimental setup for measuring the tensile strengths of AZO-CLCP films or AZO-CLCP/CNT nanocomposite films by using the universal material mechanical analyzer. The tensile rate was 1 mm·min^−1^, Figure S4: Stress-strain curves of F10(a), F60(b) and CF60(c) measured with the tensile direction of being parallel (1) and perpendicular (2) to the alignment direction, Movie S1: The microvalve membrane actuator performs opening or closing the valve outlet under switching on/off of UV-light.

## References

[1] Jiang H.R., Li C.S., Xue Z.H. (2013). Actuators based on liquid crystalline elastomer materials. Nanoscale.

[2] Yu H.F. (2014). Photoresponsive liquid crystalline block copolymers: From photonics to nanotechnology. Prog. Polym. Sci..

[3] Gu W., Wei J., Yu Y.L. (2016). Thermo- and photo-driven soft actuators based on crosslinked liquid crystalline polymers. Chin. Phys. B.

[4] Ohm C., Brehmer M., Zentel R. (2010). Liquid crystalline elastomers as actuators and sensors. Adv. Mater..

[5] Yu H.F., Ikeda T. (2011). Photocontrollable liquid-crystalline actuators. Adv. Mater..

[6] Pang X., Xu B., Qing X., Wei J., Yu Y. (2018). Photo-induced bending behavior of post-crosslinked liquid crystalline polymer/polyurethane blend films. Macromol. Rapid Commun..

[7] Ube T., Yoda T., Ikeda T. (2018). Fabrication of photomobile polymer materials with phase-separated structure of crosslinked azobenzene liquid-crystalline polymer and poly(dimethylsiloxane). Liq. Cryst..

[8] Yue Y.F., Norikane Y., Azumi R., Koyama E. (2018). Light-induced mechanical response in crosslinked liquid-crystalline polymers with photoswitchable glass transition temperatures. Nat. Commun..

[9] Ube T., Ikeda T. (2014). Photomobile polymer materials with crosslinked liquid-crystalline structures: Molecular design, fabrication, and functions. Angew. Chem. Int. Ed..

[10] White T.J., Broer D.J. (2015). Programmable and adaptive mechanics with liquid crystal polymer networks and elastomers. Nat. Mater..

[11] Yu H.F. (2014). Recent advances in photoresponsive liquid crystalline polymers containing azobenzene chromophores. J. Mater. Chem. C.

[12] Yu Y.L., Nakano M., Ikeda T. (2003). Photomechanics: directed bending of a polymer film by light. Nature.

[13] Lee K.M., Smith M.L., Koerner H., Tabiryan N., Vaia R.A., Bunning T.J., White T.J. (2011). Photodriven, flexural-torsional oscillation of glassy azobenzene liquid crystal polymer networks. Adv. Funct. Mater..

[14] Wie J.J., Lee K.M., Smith M.L., Vaia R.A., White T.J. (2013). Torsional mechanical responses in azobenzene functionalized liquid crystalline polymer networks. Soft Matter.

[15] Tang R., Liu Z., Xu D., Liu J., Yu L., Yu H. (2015). Optical pendulum generator based on photomechanical liquid-crystalline actuators. ACS Appl. Mater. Interfaces.

[16] Yamada M., Kondo M., Mamiya J., Yu Y., Kinoshita M., Barrett C.J., Ikeda T. (2008). Photomobile polymer materials: Towards light-driven plastic motors. Angew. Chem. Int. Ed..

[17] Yamada M., Kondo M., Miyasato R., Naka Y., Mamiya J., Kinoshita M., Shishido A., Yu Y.L., Barrett C.J., Ikeda T. (2009). Photomobile polymer materials-various three-dimensional movements. J. Mater. Chem..

[18] Cheng F.T., Yin R.Y., Zhang Y.Y., Yen C.-C., Yu Y.L. (2010). Fully plastic microrobots which manipulate objects using only visible light. Soft Matter.

[19] Huang C.L., Lv J.-A., Tian X.J., Wang Y.C., Yu Y.L., Liu J. (2015). Miniaturized swimming soft robot with complex movement actuated and controlled by remote light signals. Sci. Rep..

[20] Wani O.M., Zeng H., Priimagi A. (2017). A light-driven artificial flytrap. Nat. Commun..

[21] Chen M., Xing X., Liu Z., Zhu Y., Liu H., Yu Y., Cheng F. (2010). Photodeformable polymer material: Towards light-driven micropump applications. Appl. Phys. A.

[22] Chen M.L., Huang H.T., Zhu Y.T., Liu Z., Xing X., Cheng F.T., Yu Y.L. (2011). Photodeformable CLCP material: study on photo-activated microvalve applications. Appl. Phys. A.

[23] Van Oosten C.L., Bastiaansen C.W.M., Broer D.J. (2009). Printed artificial cilia from liquid-crystal network actuators modularly driven by light. Nat. Mater..

[24] Lv J.A., Liu Y.Y., Wei J., Chen E.Q., Qin L., Yu Y.L. (2016). Photocontrol of fluid slugs in liquid crystal polymer microactuators. Nature.

[25] Wu W., Yao L., Yang T., Yin R., Li F., Yu Y. (2011). NIR-light-induced deformation of cross-linked liquid-crystal polymers using upconversion nanophosphors. J. Am. Chem. Soc..

[26] Jiang Z., Xu M., Li F., Yu Y. (2013). Red-light-controllable liquid-crystal soft actuators via low-power excited upconversion based on triplet-triplet annihilation. J. Am. Chem. Soc..

[27] Yu L., Cheng Z., Dong Z., Zhang Y., Yu H. (2014). Photomechanical response of polymer-dispersed liquid crystals/graphene oxide nanocomposites. J. Mater. Chem. C.

[28] Cheng Z.X., Wang T.J., Li X., Zhang Y.H., Yu H.F. (2015). NIR-Vis-UV light-responsive actuator films of polymer-dispersed liquid crystal/graphene oxide nanocomposites. ACS Appl. Mater. Interf..

[29] Li C.S., Huang X.Z., Jiang H.R. (2015). Reversible Photo Actuated Composite Bulk with Nematic Liquid Crystalline Elastomer Matrix. Mol. Cryst. Liq. Cryst..

[30] Zou W.Q., Huang X.Z., Li Q.K., Guo L.C., Li C.S., Jiang H.R. (2016). Photo-thermo-mechanically actuated liquid crystalline elastomer nanocomposite reinforced by polyurethane fiber-network. Mol. Cryst. Liq. Cryst..

[31] Sun X.M., Sun H., Li H.P., Peng H.S. (2013). Developing polymer composite materials: carbon nanotubes or graphene?. Adv. Mater..

[32] Yang L., Setyowati K., Li A., Gong S., Chen J. (2008). Reversible infrared actuation of carbon nanotube-liquid crystalline elastomer nanocomposites. Adv. Mater..

[33] Ji Y., Huang Y.Y., Rungsawang R., Terentjev E.M. (2010). Dispersion and alignment of carbon nanotubes in liquid crystalline polymers and elastomers. Adv. Mater..

[34] Li C., Liu Y., Lo C.-W., Jiang H. (2011). Reversible white-light actuation of carbon nanotube incorporated liquid crystalline elastomer nanocomposites. Soft Matter.

[35] Li C., Liu Y., Huang X., Jiang H. (2012). Direct sun-driven artificial heliotropism for solar energy harvesting based on a photo-thermomechanical liquid-crystal elastomer nanocomposite. Adv. Funct. Mater..

[36] Kohlmeyer R.R., Chen J. (2013). Wavelength-selective, IR light-driven hinges based on liquid crystalline elastomer composites. Angew. Chem. Int. Ed..

[37] Yang Y.K., Zhan W.J., Peng R.G., He C.G., Pang X.C., Shi D., Jiang T., Lin Z.Q. (2015). Graphene-Enabled Superior and Tunable Photomechanical Actuation in Liquid Crystalline Elastomer Nanocomposites. Adv. Mater..

[38] Li C.S., Liu Y., Huang X.Z., Li C.H., Jiang H.R. (2015). Light actuation of graphene-oxide incorporated liquid crystalline elastomer nanocomposites. Mol. Cryst. Liq. Cryst..

[39] Liu W., Guo L.X., Lin B.P., Zhang X.Q., Sun Y., Yang H. (2016). Near-infrared responsive liquid crystalline elastomers containing photothermal conjugated polymers. Macromolecules.

[40] Wang M., Sayed S.M., Guo L.X., Lin B.P., Zhang X.Q., Sun Y., Yang H. (2016). Multi-stimuli responsive carbon nanotube incorporated polysiloxane azobenzene liquid crystalline elastomer composites. Macromolecules.

[41] Wang Y.C., Huang X.Z., Zhang J.Q., Bi M., Zhang J.D., Niu H.Y., Li C.S., Yu H.F., Wang B.S., Jiang H.R. (2017). Two-step crosslinked liquid-crystalline elastomer with reversible two-way shape memory characteristics. Mol. Cryst. Liq. Cryst..

[42] Wang W., Sun X.M., Wu W., Peng H.S., Yu Y.L. (2012). Photoinduced deformation of crosslinked liquid-crystalline polymer film oriented by a highly aligned carbon nanotube sheet. Angew. Chem. Int. Ed..

[43] Sun X.M., Wang W., Qiu L.B., Guo W.H., Yu Y.L., Peng H.S. (2012). Unusual reversible photomechanical actuation in polymer/nanotube composites. Angew. Chem. Int. Ed..

[44] Li C.S., Lo C.-W., Zhu D.F., Li C.H., Liu Y., Jiang H.R. (2009). Synthesis of a photoresponsive liquid-crystalline polymer containing azobenzene. Macromol. Rapid Commun..

[45] Moon J., Choi J., Cho M. (2017). Opto-mechanical behavior and interfacial characteristics of crosslinked liquid crystalline polymer composites with carbon nanotube fillers. Carbon.

[46] Barrett C.J., Mamiya J.I., Yager K.G., Ikeda T. (2007). Photo-mechanical effects in azobenzene-containing *soft* materials. Soft Matter.

