# Supplementary material for: Photo Actuation Performance of Nanotube Sheet Incorporated Azobenzene Crosslinked Liquid Crystalline Polymer Nanocomposite
Source: Polymers (Basel). 2019 Apr 23;11(4):735. doi: 10.3390/polym11040735 (PMC6523392; doi:10.3390/polym11040735)
Supplement: Supplementary file 1 [file polymers-11-00735-s001.zip › polymers-433595-supplementary/support information polymers-433595.pdf]

Support Information

Photo Actuation Performance of Nanotube Sheet Incorporated Azobenzene Crosslinked Liquid Crystalline Polymer Nanocomposite

Movie S1: The microvalve membrane actuator performs opening or closing the valve outlet under switching on/off of UV-light.

^{1}H NMR measurement data:

The ^{1}H NMR measurement of DA11AB: δ 1.4-1.9(m, 36H), 3.9(t, 4H), 4.1(t, 4H), 5.8(dd, 2H), 6.1(dd, 2H), 6.4(dd, 2H), 6.9(m, 4H), 7.8(m, 4H). The ^{1}H NMR measurement of C9A: δ 1.35-1.85(m, 28H), 2.24(s, 3H), 4.05(t, J=6.5 Hz, 4H), 4.18(t, J=6.5 Hz, 4H), 5.74(dd, J=2, 10 Hz, 2H), 6.07(dd, J=10, 7 Hz, 2H), 6.33(dd, J=2, 17 Hz, 2H), 6.95-6.99(m, 4H), 7.12-7.36(m, 3H), 8.12-8.17(m， 4H).

Mass spectra (MS) data:

The mass fraction of the element "C", "H" and "N" obtained from MS measurement of DA11AB was 72.29%, 8.80% and 4.48% respective, which are closed to the theoretical value of 72.47%, 8.82% and 4.23% respective. In addition, the mass fraction of the element "C" and "H" obtained from MS measurement of C9A was 71.68% and 7.48% respective, which are closed to the theoretical value of 71.43% and 7.41% respective.

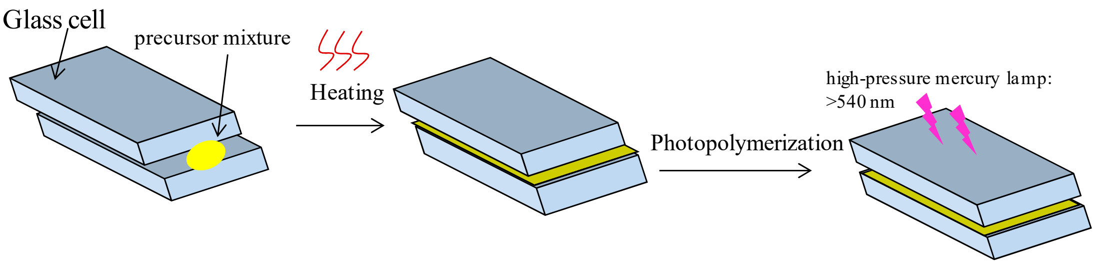

Figure S1. A schematic illustration of the preparation process of AZO-CLCP film.

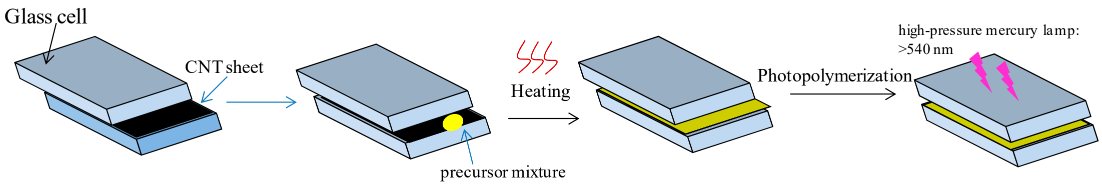

Figure S2. A schematic illustration of the preparation process of AZO-CLCP/CNT film.

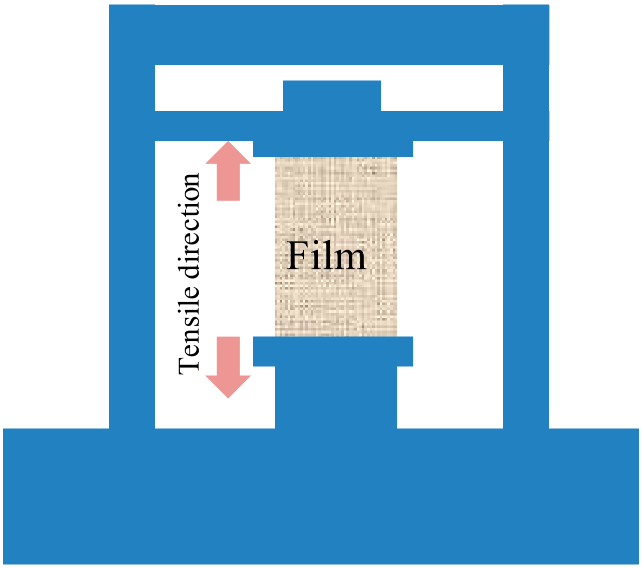

Figure S3. Schematic illustration of the experimental setup for measuring the tensile strengths of AZO-CLCP films or AZO-CLCP/CNT nanocomposite films by using the universal material mechanical analyzer. The tensile rate was 1 mm min^{-1}.

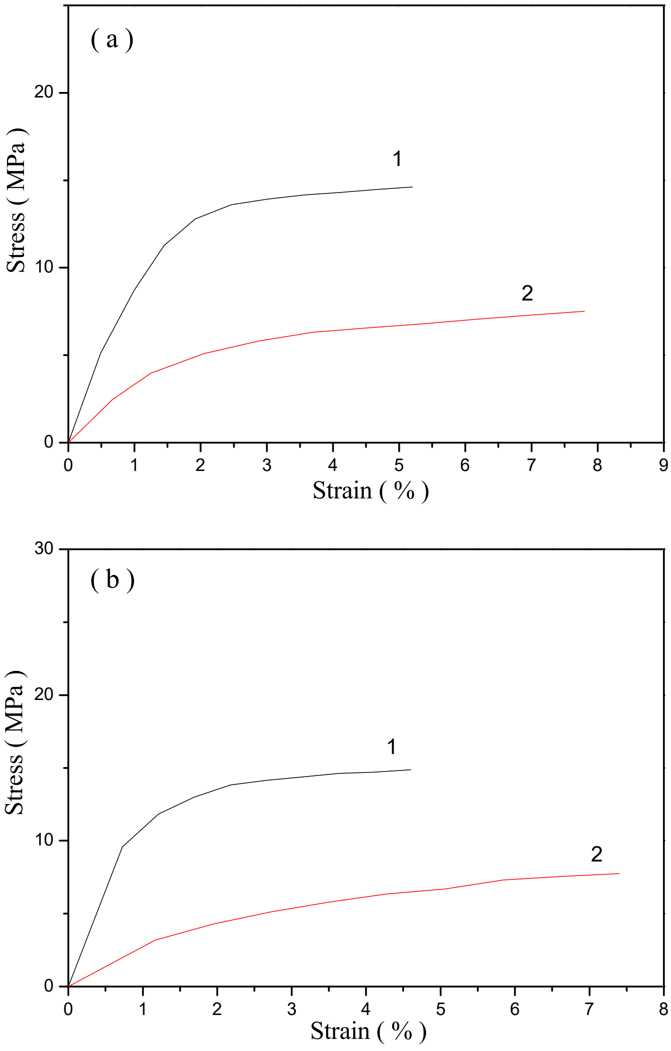

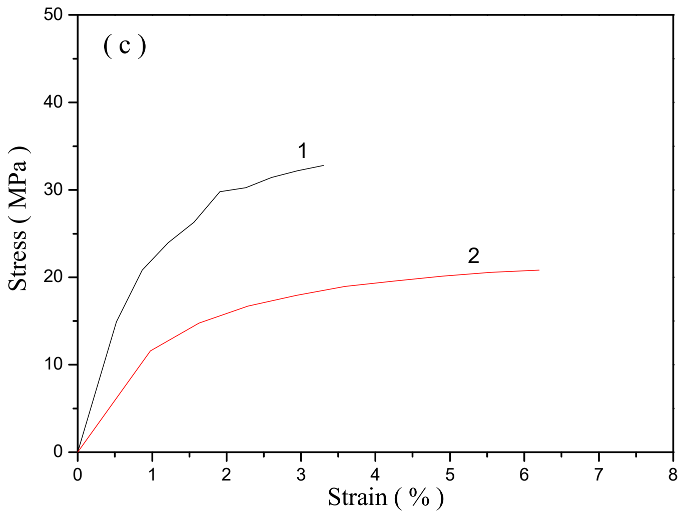

Figure S4. Stress-strain curves of F10(a), F60(b) and CF60(c) measured with the tensile direction of being parallel (1) and perpendicular (2) to the alignment direction.
